# Associations of Triglycerides/High-Density Lipoprotein Cholesterol Ratio With Insulin Resistance, Impaired Glucose Tolerance, and Diabetes in American Adults at Different Vitamin D3 Levels

**DOI:** 10.3389/fendo.2021.735736

**Published:** 2022-02-04

**Authors:** Yuanyuan Liu, Rongpeng Gong, Gang Luo, Jinxia Li, Qidan Li, Lixin Yang, Xiaoxing Wei

**Affiliations:** ^1^Medical College of Qinghai University, Xining, China; ^2^Endocrinology Department, Qinghai Provincial People’s Hospital, Xining, China; ^3^College of Eco-environmental Engineering, Qinghai University, Xining, China

**Keywords:** TG/HDL ratio, diabetes mellitus, insulin resistance, impaired glucose tolerance (IGT), cross-sectional study

## Abstract

**Background:**

Previous studies have shown that vitamin D3 (VD3) may be a protective factor for diabetes mellitus (DM), while triglycerides/high-density lipoprotein (TG/HDL) may be a risk factor for diabetes. However, no existing study has elucidated the interaction between TG/HDL and VD3. Therefore, this work aimed to investigate the relationships of TG/HDL with insulin resistance (IR), impaired glucose tolerance (IGT), and DM at different VD3 levels.

**Methods:**

With the use of the data from five National Health and Nutrition Examination Survey (NHANES) cycles, a total of 2,929 males and 3,031 females were divided into 4 groups according to their VD3 levels. Logistic regression was performed to observe the associations of TG/HDL ratio with IR, IGT, and DM in different groups.

**Results:**

The relationships of TG/HDL with IR, IGT, and DM showed a threshold effect, with the cutoff values of 1.094, 1.51, and 1.11, respectively. On both sides of the cutoff values, the correlation was first weakened and then enhanced with the increase in VD3 levels.

**Conclusion:**

TG/HDL is a risk factor for IR, IGT, and DM. Both too low and too high levels of VD3 can strengthen this association, whereas keeping VD3 at a reasonable level helps to reduce the associations of TG/HDL with IR, IGT, and DM.

## 1 Background

Vitamin D3 (VD3), also called cholecalciferol, is a type of vitamin D (1). VD3 is the precursor of hormones, which have been recently found to participate in numerous regulatory responses in the body (2–4). Studies have shown that VD3 plays a role not only in bone metabolism but also in insulin resistance (IR), impaired glucose tolerance (IGT), and diabetes mellitus (DM) (3–6).

IR is identified as an impaired response to insulin of target tissues and the resulting reduced efficiency of glucose uptake and utilization (7). If left uncontrolled, IR will develop into IGT and even DM. After feeding rats with a high-fat diet (HFD) for 12 weeks, Zhang et al. found that IR appeared in 93.3% of the rats (8). With the dramatic changes in people’s living conditions, the morbidity of DM shows an increasing trend in various countries worldwide (9–11). According to the Diabetes Atlas 2019 published by the International Diabetes Federation (IDF), there are 425 million patients with type 2 DM (T2DM) around the world (12). According to the current development trend, it is estimated that there will be 629 million DM patients aged 20–79 by the year 2045, accounting for 10% of the overall population (13). Besides, there will be more patients with IGT and IR.

At present, VD3 and triglycerides/high-density lipoprotein (TG/HDL) are identified as the factors related to IR, IGT, and DM (14–17). However, few existing studies have investigated whether the relationship between TG/HDL ratio and abnormal glucose metabolism is affected by different VD3 levels. Herein, a retrospective analysis was conducted based on the American National Health and Nutrition Examination Survey (NHANES) database, aiming to discover the difference (or evidence) in the associations of TG/HDL ratio with IR, IGT, and DM at different VD3 levels. The findings in this study will make a significant contribution to exploring the clinical prevention and treatment of DM.

## 2 Methods

### 2.1 Research Population and Test Methods

Altogether, 49,696 participants from five periods were selected from the NHANES database from 2009 to 2018. The NHANES project is a subject study strictly formulated by the National Center for Health Statistics (NCHS) to meet the different population representations. NHANES is a persistent project that makes 2 years as a period, and it ensures that the sample is representative of the American population through multilevel and complex sampling design. There are about 5,000 people who receive the sampling survey, which covers diverse aspects like population, social, economy, diet, and health. The laboratory examination section includes medical and physiological tests. All data are collected by professional and trained personnel. NHANES follows a strict standard and protocol to ensure the privacy of each participant, and the information is not used for identification under the U.S. federal law as well.

In this study, strict inclusion and exclusion criteria were applied to select eligible participants. To be specific, participants meeting one or more of the following criteria were excluded: 1) those aged under 18 years; 2) those with no key indicators of insulin, fasting glucose, TG, or HDL; and 3) others (including patients taking drugs that affected blood lipid metabolism, glucose metabolism, and parathyroid metabolism; patients suffering from immunodeficiency, infectious diseases, or malignant tumors; and patients with a recent history of surgery, trauma, severe infection or other stress).

**Figure 1:**
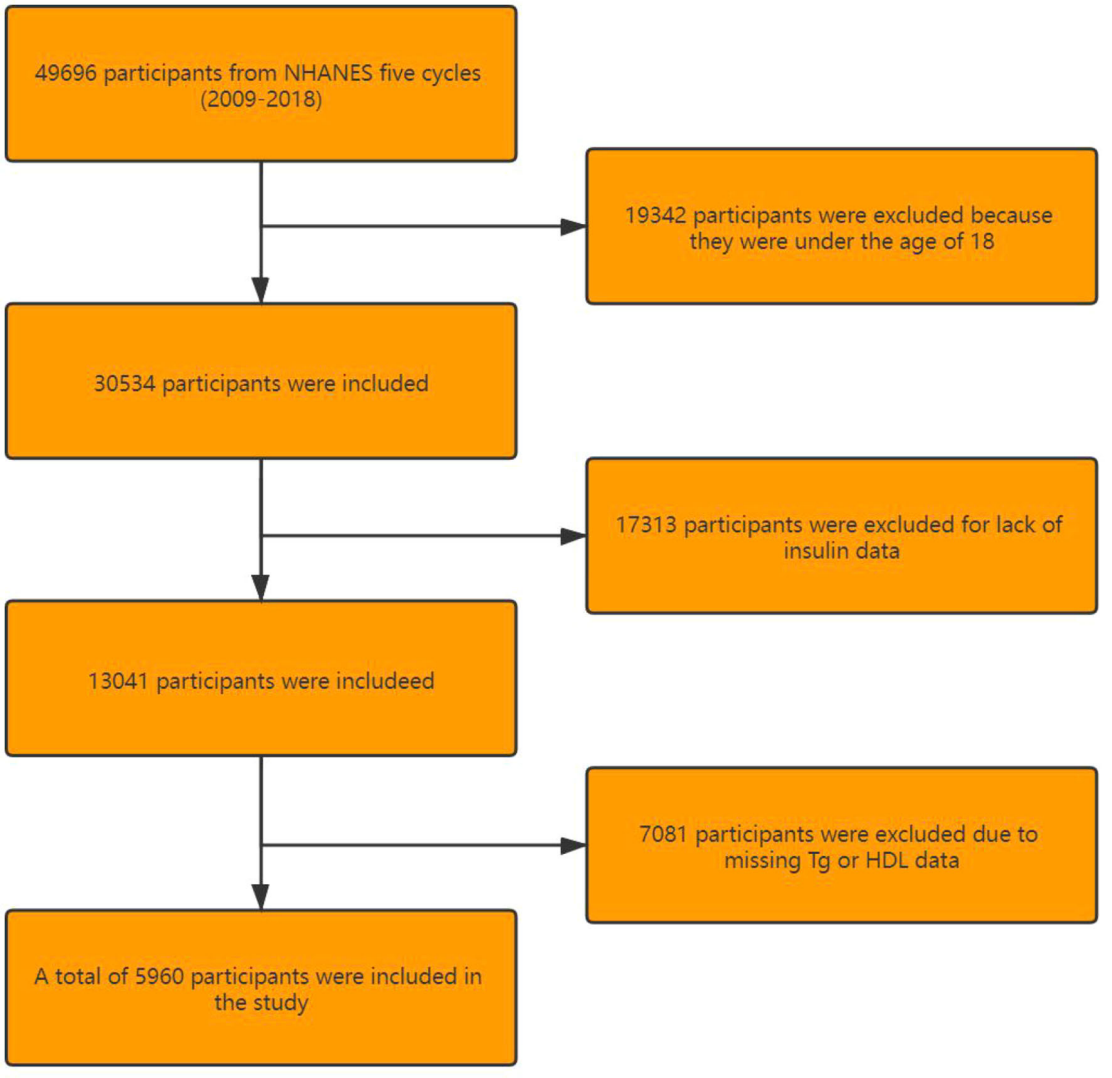
Flowchart of participant selection.

NHANES 2009–2018 covered five periods (2009–2010, 2011–2012, 2013–2014, 2015–2016, and 2017–2018), involving 49,696 participants in total. Among them, 19,342 participants were excluded due to the age of <18 years, 17,313 because of the unavailability of insulin data, 3,374 due to unavailable TG data, and 3,707 because of the lack of HDL data. Finally, 5,960 participants were included in this trial (**Figure 1**).

Ultrahigh-performance liquid chromatography–tandem mass spectrometry (UHPLC-ms/MS) was performed to quantify VD3 levels in human serum samples. After fasting for 9 h, the fasting plasma glucose (FPG) and insulin levels in the enrolled participants were measured *via* venipuncture in the morning. Blood lipid data were provided by the laboratory at the University of Minnesota.

**Figure 2:**
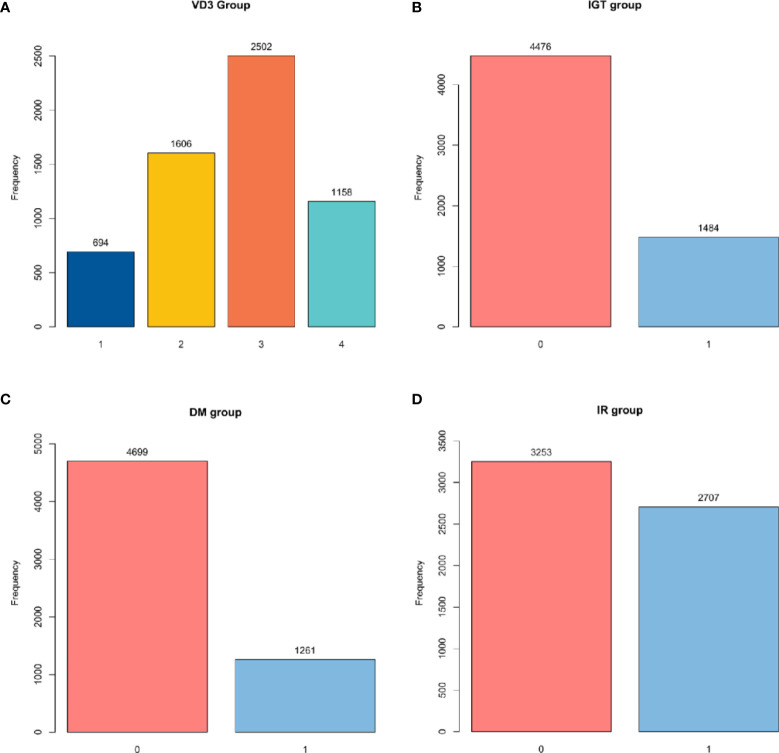
Group diagram of each group. **(A)** Number of participants in different VD3 groups. **(B)** Number of participants with or without IGT. **(C)** Number of participants with or without DM. **(D)** Number of participants with or without IR. VD3, vitamin D3; IGT, impaired glucose tolerance; DM, diabetes mellitus; IR, insulin resistance.

In this study, TG/HDL ratio was used as the independent variable, whereas IR, IGT, and DM were used as the dependent variables. According to the definition of VD3, participants were divided into four groups, including VD3 deficiency group (N1, VD3 < 30 ng/ml), VD3 insufficient group (N2, 30 ng/ml < VD3 ≤ 50 ng/ml), VD3 moderate group (N3, 50 ng/ml < VD3 ≤ 80 ng/ml), and overdose group (N4, VD3 ≥ 80 ng/ml) (**Figure 2**).

### 2.2 Disease Determination

In this study, the disease determination criterion was strictly formulated based on the international standard.

#### 2.2.1 Insulin Resistance

The clinical definition of IR remains elusive, as no generally accepted test is available for IR (18). In some studies, IR is defined by the steady-state model evaluation formula: fasting glucose insulin (μU/ml) × fasting glucose (mmol/L)/22.5 (19). In other research, IR > 2.6 is regarded as IR in a normal American population, which was taken as the determination criterion in our study (**Figure 2**).

#### 2.2.2 Impaired Glucose Tolerance

In this study, according to relevant questionnaires and laboratory tests, IGT was defined as self-reported DM or FPG ≥ 6.0 mmol/L (type 1 DM, gestational DM, and specific types of DM were excluded) (20) (**Figure 2**).

#### 2.2.3 Diabetes Mellitus

DM is defined based on the American Diabetes Association Standards 2015. In this study, according to relevant questionnaires and laboratory tests, DM was defined as self-reported DM or glycosylated hemoglobin ≥6.5% and FPG ≥ 6.0 mmol/L (type 1 DM, gestational DM, and specific types of DM were excluded) (21) (**Figure 2**).

#### 2.2.4 Hypertension

In the present work, blood pressure (BP) was measured thrice while the participants were at rest, and the three measurements were averaged to assess whether the participants had hypertension or not. Typically, hypertension was defined as systolic BP (SBP) ≥140 mmHg and/or diastolic BP (DBP) ≥90 mmHg or self-reported hypertension and use of antihypertensive drugs. The definition conformed to the American Heart Association Blood Pressure Guidelines 2017 (22).

#### 2.2.5 Smoking

Participants were classified into three groups according to their different smoking conditions, namely, 1) current smokers (who smoked at least one cigarette a day in the past 30 days), 2) current non-smokers (who smoked an average of less than 1 cigarette per day in the past 30 days or more than 100 cigarettes in their lifetime), and 3) non-smokers (who reported that they smoked less than 100 cigarettes in their lifetime or never smoked). In this study, due to the small number of non-smokers, the current non-smokers and non-smokers were finally combined as non-smokers (23).

#### 2.2.6 Alcohol Use

After the classification of alcohol consumption in previous studies was checked, the participants were finally divided into two groups, including drinkers (who drank more than 12 drinks a year) and non-drinkers (who drank no more than 12 drinks a year) (24).

#### 2.2.7 Income and Education

In this study, participants earning more than $100,000 were defined as high income, while those with a junior college education or higher were defined as high education.

## 3 Statistical Analysis

The NHANES database selects an annual of 5,000 people from a framework of 15 different locations in all counties of the United States to ensure that the data are universal and widespread. The unique multistage probability sampling technique employed by the NHANES database enables the data to better represent the incidence of IR in the U.S. population over the past few years. In this study, samples were collected from the NHANES database at five consecutive periods to make this study more convincing. Besides, data were selected through rigorous comprehensive screening to obtain a more representative sample.

Statistical analysis was performed using R language version 4.0.2, and a two-sided *p*-value <0.05 indicated statistical significance. Multivariate logistic regression was adopted to analyze the associations of TG/HDL ratio with IR, IGT, and DM, under different VD3 levels in the American adult population. Continuous variables were represented by detailed sample descriptions. Classification variables were expressed as counts and weighted percentages. In addition, the skewed distribution was based on the median and Q1–Q3, whereas the normal distribution was described by the median and SD. Four multivariate logistic regression models and smooth fitting curves were established to analyze the relationships of TG/HDL ratio with IR, IGT, and DM at different VD3 levels. Moreover, two-stage logistic regression and a log-likelihood ratio test were performed to analyze whether there was a non-linear relationship. Multiple imputations were utilized to compensate for the missing variables in this study. To increase the statistical power and avoid bias, the missing data of covariates were removed from this analysis. A sensitivity analysis was also performed to evaluate whether the generated data differed considerably from the raw data. According to sensitivity analysis, the generated complete data were similar to the raw data. As a result, our following multivariable analyses were carried out using the raw data based on Rubin’s guidelines.

### 3.1 Selection of Covariates

In this study, covariates were screened according to the following criteria, 1) baseline characteristics of the population; 2) variables affecting TG/HDL, IR, IGT, and DM identified in previous studies; 3) the basic model changed by more than 10% after introducing covariates; and 4) experience gained in clinical work.

In line with the abovementioned criteria, gender, age, race, alanine aminotransferase (ALT), aspartate aminotransferase (AST), TG, total cholesterol (TC), γ-glutamyl transpeptidase (GGT), albumin (ALB), HDL, smoking, alcohol use, income, and education were selected as the covariates.

## 4 Results

### 4.1 Characteristics of the Participants

A total of 5,960 participants were finally included according to the strict inclusion and exclusion criteria, including 2,929 males (49.1%) and 3,031 females (50.9%). Among these samples, there were significant differences in variables of gender, age, race, smoking, education, hyperuricemia, hypertension, hypercholesterolemia, body mass index (BMI), waist, IR, DM, IGT, ALT, AST, alkaline phosphatase (ALP), total bilirubin (TBIL), blood urea nitrogen (BUN), and GGT (*p* < 0.001) among VD3 groups (N1–N4). Notably, the highest proportions of smoking (79.1%), alcohol use (34.3%), IR (55.6%), IGT (31.7%), and DM (26.9%), together with the highest values of BMI (30.6 ± 8.8) and waist (101.2 ± 20.8) were observed in the N1 group. By contrast, the highest proportions of education (61.3%), income (45.3%), hyperuricemia (23.1%), hypertension (77.0%), and hypercholesterolemia (54.8%), as well as the highest values of AST (23.0, (20.0, 27.0)) and BUN (4.6, (3.9, 6.1)), were seen in the N4 group. The average age of the total samples was 43.0 ± 20.6 years, and the oldest age (52.8 ± 19.9) was observed in the N4 group (**Table 1**).

**Table 1:** Basic characteristics of the participants.

Variables	Total (n = 5,960)	N1 (n = 694)	N2 (n = 1,606)	N3 (n = 2,502)	N4 (n = 1,158)	p-Value
Gender, n (%)						< 0.001
Male	2,929 (49.1)	305 (43.9)	786 (48.9)	1,376 (55)	462 (39.9)	
Female	3,031 (50.9)	389 (56.1)	820 (51.1)	1,126 (45)	696 (60.1)	
Age (mean ± SD)	43.0 ± 20.6	41.2 ± 18.9	38.5 ± 19.3	41.9 ± 20.7	52.8 ± 19.9	<0.001
Race (n (%))						<0.001
Mexican American	804 (13.5)	89 (12.8)	299 (18.6)	350 (14)	66 (5.7)	
Other Hispanics	576 (9.7)	36 (5.2)	156 (9.7)	297 (11.9)	87 (7.5)	
Non-Hispanic white	2,331 (39.1)	116 (16.7)	357 (22.2)	1,096 (43.8)	762 (65.8)	
Non-Hispanic black	1,311 (22.0)	342 (49.3)	497 (30.9)	370 (14.8)	102 (8.8)	
Other race	938 (15.7)	111 (16)	297 (18.5)	389 (15.5)	141 (12.2)	
Alcohol use (n (%))						0.095
No	4,113 (69.0)	456 (65.7)	1,082 (67.4)	1,762 (70.4)	813 (70.2)	
Yes	1,842 (30.9)	238 (34.3)	522 (32.5)	738 (29.5)	344 (29.7)	
SMOK (n (%))						<0.001
No	1,786 (30.0)	145 (20.9)	448 (27.9)	761 (30.4)	432 (37.3)	
Yes	4,174 (70.0)	549 (79.1)	1,158 (72.1)	1,741 (69.6)	726 (62.7)	
EDU (n (%))						<0.001
No higher education	2,668 (44.8)	339 (48.8)	749 (46.6)	1,132 (45.2)	448 (38.7)	
Higher education	3,292 (55.2)	355 (51.2)	857 (53.4)	1,370 (54.8)	710 (61.3)	
INCOME (n (%))						<0.001
No more than $100,000	3,657 (61.4)	495 (71.3)	1,037 (64.6)	1,492 (59.6)	633 (54.7)	
More than $100,000	2,303 (38.6)	199 (28.7)	569 (35.4)	1,010 (40.4)	525 (45.3)	
HUA (n (%))						<0.001
No	4,849 (81.4)	536 (77.2)	1,307 (81.4)	2,116 (84.6)	890 (76.9)	
Yes	1,111 (18.6)	158 (22.8)	299 (18.6)	386 (15.4)	268 (23.1)	
Hbp (n (%))						<0.001
No	1,889 (31.7)	181 (26.1)	581 (36.2)	861 (34.4)	266 (23)	
Yes	4,071 (68.3)	513 (73.9)	1,025 (63.8)	1,641 (65.6)	892 (77)	
HTC (n (%))						<0.001
No	3,405 (57.1)	408 (58.8)	1,024 (63.8)	1,450 (58)	523 (45.2)	
Yes	2,555 (42.9)	286 (41.2)	582 (36.2)	1,052 (42)	635 (54.8)	
BMI, (mean ± SD)	28.0 ± 7.1	30.6 ± 8.8	28.5 ± 7.4	27.4 ± 6.5	27.1 ± 6.3	<0.001
Waist (mean ± SD)	95.8 ± 17.7	101.2 ± 20.8	96.1 ± 18.2	94.5 ± 16.9	94.8 ± 15.8	<0.001
Insulin resistance (n (%))						<0.001
No	3,253 (54.6)	308 (44.4)	780 (48.6)	1,407 (56.2)	758 (65.5)	
Yes	2,707 (45.4)	386 (55.6)	826 (51.4)	1,095 (43.8)	400 (34.5)	
Diabetes (n (%))						<0.001
No	4,699 (78.8)	507 (73.1)	1,257 (78.3)	2,036 (81.4)	899 (77.6)	
Yes	1,261 (21.2)	187 (26.9)	349 (21.7)	466 (18.6)	259 (22.4)	
Prediabetes (n (%))						<0.001
No	4,476 (75.1)	474 (68.3)	1,211 (75.4)	1,945 (77.7)	846 (73.1)	
Yes	1,484 (24.9)	220 (31.7)	395 (24.6)	557 (22.3)	312 (26.9)	
ALT (median (IQR))	19.0 (15.0, 26.0)	19.0 (14.0, 25.0)	19.0 (15.0, 27.0)	20.0 (15.0, 27.0)	20.0 (16.0, 25.0)	0.017
AST (median (IQR))	22.0 (19.0, 27.0)	21.0 (18.0, 26.0)	22.0 (19.0, 26.0)	22.0 (19.0, 27.0)	23.0 (20.0, 27.0)	<0.001
ALP (median (IQR))	66.0 (54.0, 84.0)	68.0 (55.0, 85.0)	68.0 (55.0, 87.0)	66.0 (54.0, 85.0)	63.0 (51.0, 77.0)	<0.001
TBIL (median (IQR))	12.0 (8.6, 13.7)	10.3 (8.6, 13.7)	10.3 (8.6, 13.7)	12.0 (8.6, 13.7)	12.0 (8.6, 13.7)	<0.001
BUN (median (IQR))	4.3 (3.2, 5.4)	3.6 (2.9, 4.6)	3.9 (3.2, 4.6)	4.3 (3.6, 5.4)	4.6 (3.9, 6.1)	<0.001
TC (median (IQR))	4.7 (4.0, 5.4)	4.6 (4.0, 5.4)	4.6 (4.0, 5.4)	4.6 (4.0, 5.4)	4.9 (4.2, 5.6)	<0.001
UA (median (IQR))	315.2 (261.7, 374.7)	324.2 (255.8, 386.6)	315.2 (255.8, 374.7)	315.2 (261.7, 368.8)	309.3 (261.7, 374.7)	0.426
GGT (median (IQR))	17.0 (13.0, 26.0)	18.0 (13.2, 31.0)	18.0 (13.0, 27.0)	17.0 (12.0, 25.0)	17.0 (13.0, 26.0)	<0.001
FPG (median (IQR))	5.2 (4.8, 5.7)	5.2 (4.8, 5.8)	5.2 (4.8, 5.7)	5.2 (4.8, 5.6)	5.2 (4.8, 5.7)	0.092
TG/HDL (median (IQR))	0.8 (0.5, 1.3)	0.8 (0.5, 1.3)	0.7 (0.5, 1.3)	0.8 (0.5, 1.4)	0.8 (0.5, 1.3)	0.071

EDU, education; SMOK, smoking; BMI, body mass index; ALT, alanine aminotransferase; ALB, albumin; AST, aspartate aminotransferase; ALP, alkaline phosphatase; BUN, blood urea nitrogen; GGT, γ-glutamyl transpeptidase; UA, uric acid; FPG, fasting plasma glucose; TC, total cholesterol; TG/HDL, triglycerides/high-density lipoprotein.

### 4.2 Univariate Logistic Analysis

**Table 2** presents the results of a univariate analysis to elucidate factors related to IR, IGT, and DM. As a result, the positive factors were age, drinking, hypertension, hyperuricemia, hypercholesterolemia, BMI, waist, TG, ALT, BUN, GGT, and TG/HDL ratio, whereas education, income, smoking, and HDL and VD3 levels were negatively correlated with IR, IGT, and DM (**Table 2**). Among the above variables, TG/HDL ratio showed the most significant relationship with IR among the diverse continuous variables, but its relations with IGT and DM were weaker (**Table 2**).

**Table 2:** Univariate analysis of IR, IGT, and DM.

Variables	IR		IGT		DM	
	OR (95% CI)	p-Value	OR (95% CI)	p-Value	OR (95% CI)	p-Value
Age	1.01 (1~1.01)	<0.001	1.06 (1.05~1.06)	<0.001	1.06 (1.06~1.06)	<0.001
**Gender**						
Male	1		1		1	
Female	0.99 (0.9~1.1)	0.89	0.79 (0.7~0.89)	<0.001	0.85 (0.75~0.96)	0.011
**Education**						
No higher education	1		1		1	
Higher education	0.8 (0.72~0.89)	<0.001	0.65 (0.58~0.74)	<0.001	0.62 (0.55~0.7)	<0.001
**Income**						
No more than $100,000	1		1		1	
More than $100,000	0.75 (0.68~0.84)	<0.001	0.74 (0.66~0.84)	<0.001	0.72 (0.63~0.82)	<0.001
**Alcohol use**						
No	1		1		1	
Yes	1.25 (1.12~1.4)	<0.001	1.15 (1.02~1.31)	0.028	1.22 (1.07~1.39)	0.003
**Smoking**						
No	1		1		1	
Yes	0.93 (0.83~1.04)	0.195	0.74 (0.65~0.84)	<0.001	0.76 (0.67~0.87)	<0.001
**Hyperuricemia**						
No	1		1		1	
Yes	2.6 (2.27~2.98)	<0.001	2.43 (2.11~2.79)	<0.001	2.32 (2.01~2.67)	<0.001
**Hypertension**						
No	1		1		1	
Yes	1.71 (1.53~1.91)	<0.001	6.56 (5.43~7.91)	<0.001	7.58 (6.1~9.42)	<0.001
**Hypercholesterolemia**						
No	1		1		1	
Yes	1.73 (1.56~1.92)	<0.001	4.14 (3.65~4.7)	<0.001	4.48 (3.91~5.13)	<0.001
BMI	1.18 (1.17~1.2)	<0.001	1.1 (1.09~1.11)	<0.001	1.09 (1.08~1.1)	<0.001
Waist	1.07 (1.06~1.07)	<0.001	1.05 (1.05~1.06)	<0.001	1.05 (1.05~1.05)	<0.001
TC	1.04 (0.99~1.09)	0.097	1.13 (1.07~1.19)	<0.001	1.11 (1.05~1.17)	<0.001
TG	2.06 (1.91~2.21)	<0.001	1.48 (1.39~1.57)	<0.001	1.44 (1.36~1.53)	<0.001
HDL	0.15 (0.13~0.18)	<0.001	0.41 (0.35~0.49)	<0.001	0.41 (0.34~0.49)	<0.001
TG/HDL	2.09 (1.94~2.25)	<0.001	1.3 (1.24~1.36)	<0.001	1.28 (1.22~1.34)	<0.001
ALT	1.02 (1.02~1.02)	<0.001	1.01 (1.01~1.01)	<0.001	1.01 (1.01~1.01)	<0.001
AST	1 (1~1.01)	0.174	1 (1~1.01)	0.005	1 (1~1.01)	0.032
GGT	1.01 (1.01~1.01)	<0.001	1.01 (1.01~1.01)	<0.001	1.01 (1.01~1.01)	<0.001
ALP	1 (1~1)	<0.001	1 (1~1)	<0.001	1 (1~1)	<0.001
BUN	1.03 (1.01~1.06)	0.009	1.32 (1.27~1.36)	<0.001	1.32 (1.27~1.36)	<0.001
UA	1.01 (1~1.01)	<0.001	1.01 (1~1.01)	<0.001	1.01 (1~1.01)	<0.001

IR, insulin resistance; BMI, body mass index; ALT, alanine aminotransferase; AST, aspartate aminotransferase; ALP, alkaline phosphatase; BUN, blood urea nitrogen; GGT, γ-glutamyl transpeptidase; UA, uric acid; TC, total cholesterol; TG, triglycerides; HDL, high-density lipoprotein.

### 4.3 Multiple Logistic Regression Models

**Figure 3:**
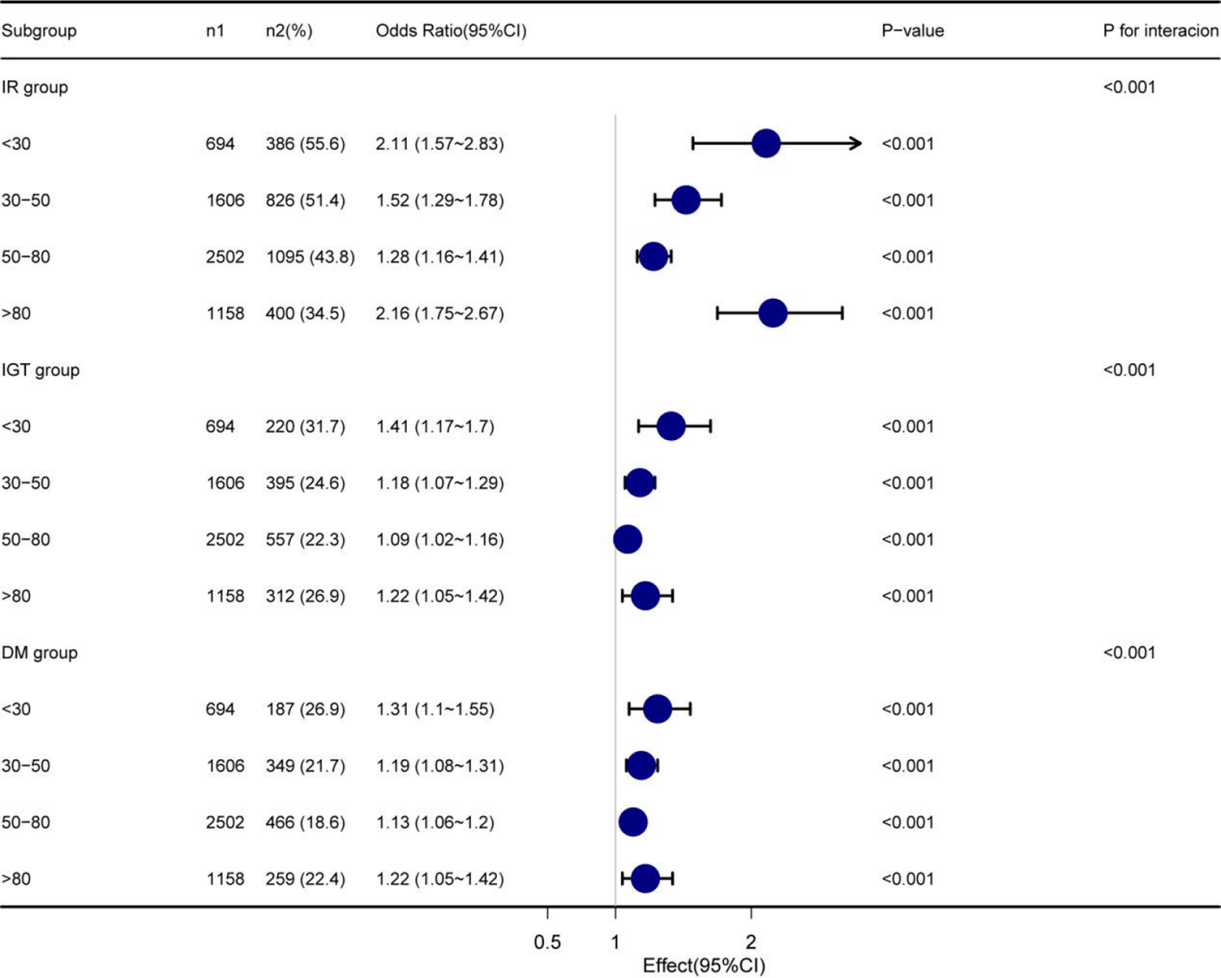
Subgroup analysis based on the multivariate logistic regression analysis of the association between TG/HDL ratio and IR, IGT, and DM. TG/HDL, triglycerides/high-density lipoprotein; IR, insulin resistance; IGT, impaired glucose tolerance; DM, diabetes mellitus.

Four models were constructed to analyze the independent effect of the TG/HDL ratio on IR, IGT, and DM at different VD3 levels. The odds ratios (ORs) and 95% CIs are shown in **Table 3**. The sizes of ORs and 95% CIs were interpreted as the corresponding changes in the incidence rates of IR, IGT, and DM with the increase in TG/HDL ratio by one SD. In the fully adjusted model (Model 4), the ORs of the association between TG/HDL ratio and IR were 2.11 (1.57~2.83), 1.52 (1.29~1.78), 1.28 (1.16~1.41), and 2.16 (1.75~2.67) in the N1–N4 groups, respectively. Meanwhile, the ORs regarding the association between TG/HDL ratio and IGT were 1.41 (1.17~1.70), 1.18 (1.07~1.29), 1.09 (1.02~1.16), and 1.22 (1.05~1.42) in the N1–N4 groups, respectively. The ORs of the association between TG/HDL ratio and DM were 1.31 (1.1~1.55), 1.19 (1.08~1.31), 1.13 (1.06~1.20), and 1.22 (1.05~1.42) in the N1–N4 groups, respectively. In addition, TG/HDL ratio was also related to VD3 levels in IR, IGT, and DM (*p* for interaction <0.001) (**Table 3** and **Figure 3**). The relatively strongest relationships of TG/HDL with IR, IGT, and DM were observed at the lowest and highest levels of VD3 groups (N1 and N4, **Table 3**).

**Table 3:** Multiple regression analysis of different VD3 levels.

Outcomes	Model 1		Model 2		Model 3		Model 4		*p* for interaction
	OR (95% CI)	p-Value	OR (95% CI)	p-Value	OR (95% CI)	p-Value	OR (95% CI)	p-Value	
IR group									<0.001
N1 (<30)	2.33 (1.83~2.96)	<0.001	2.63 (2.02~3.43)	<0.001	2.02 (1.55~2.64)	<0.001	2.11 (1.57~2.83)	<0.001	
N2 (30–50)	2.01 (1.73~2.34)	<0.001	2.23 (1.89~2.63)	<0.001	1.52 (1.31~1.78)	<0.001	1.52 (1.29~1.78)	<0.001	
N3 (50–80)	1.87 (1.68~2.07)	<0.001	1.88 (1.69~2.09)	<0.001	1.31 (1.18~1.44)	<0.001	1.28 (1.16~1.41)	<0.001	
N4 (>80)	3.27 (2.69~3.98)	<0.001	3.22 (2.63~3.94)	<0.001	2.23 (1.81~2.74)	<0.001	2.16 (1.75~2.67)	<0.001	
IGT group									<0.001
N1 (<30)	1.54 (1.33~1.8)	<0.001	1.63 (1.36~1.94)	<0.001	1.46 (1.21~1.75)	<0.001	1.41 (1.17~1.70)	<0.001	
N2 (30–50)	1.31 (1.19~1.43)	<0.001	1.29 (1.17~1.43)	<0.001	1.18 (1.08~1.3)	<0.001	1.18 (1.07~1.29)	0.001	
N3 (50–80)	1.18 (1.11~1.26)	<0.001	1.19 (1.1~1.27)	<0.001	1.08 (1.01~1.15)	0.016	1.09 (1.02~1.16)	0.007	
N4 (>80)	1.6 (1.39~1.84)	<0.001	1.59 (1.37~1.85)	<0.001	1.28 (1.1~1.48)	0.001	1.22 (1.05~1.42)	0.009	
DM group									<0.001
N1 (<30)	1.43 (1.24~1.65)	<0.001	1.51 (1.27~1.78)	<0.001	1.37 (1.15~1.62)	<0.001	1.31 (1.1~1.55)	0.002	
N2 (30–50)	1.29 (1.18~1.41)	<0.001	1.31 (1.19~1.44)	<0.001	1.2 (1.09~1.32)	<0.001	1.19 (1.08~1.31)	<0.001	
N3 (50–80)	1.18 (1.1~1.26)	<0.001	1.22 (1.13~1.32)	<0.001	1.11 (1.04~1.2)	0.002	1.13 (1.06~1.20)	<0.001	
N4 (>80)	1.51 (1.32~1.73)	<0.001	1.53 (1.31~1.78)	<0.001	1.27 (1.09~1.47)	0.002	1.22 (1.05~1.42)	0.01	

Model 1: non-adjusted. Model 2: adjusted for gender, age, and race. Model 3: Model 2 + adjusted for ALT, AST, TG, TC, GGT, ALB, and HDL. Model 4: Model 3 + adjusted for smoking, alcohol use, income, and education.

VD3, vitamin D3; IR, insulin resistance; IGT, impaired glucose tolerance; DM, diabetes mellitus; ALT, alanine aminotransferase; AST, aspartate aminotransferase; TG, triglycerides; TC, total cholesterol; GGT, γ-glutamyl transpeptidase; ALB, albumin; HDL, high-density lipoprotein.

### 4.4 Non-Linear Relationships

**Figure 4:**
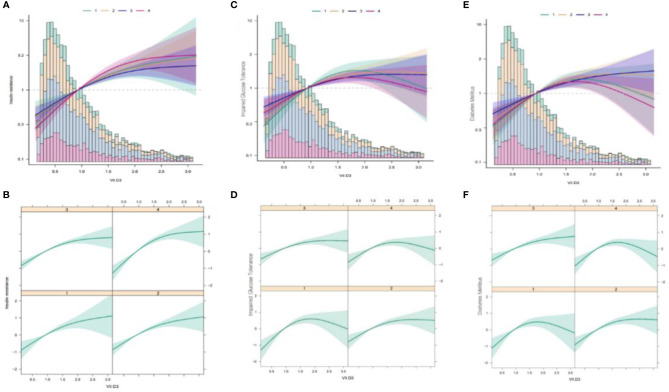
Multivariate logistic regression analysis of the associations of TG/HDL ratio with IR, IGT, and DM in different VD3 groups. TG/HDL, triglycerides/high-density lipoprotein; IR, insulin resistance; IGT, impaired glucose tolerance; DM, diabetes mellitus; VD3, vitamin D3. **(A)** The association between TG/HDL and IR at different VD3 levels (Total). **(B)** The association between TG/HDL and IR at different vD3 levels (Separate). **(C)** The association between TG/HDL and IGT at different VD3 levels (Total). **(D)** The association between TG/HDL and IGT at different vD3 levels (Separate). **(E)** The association between TG/HDL and DM at different VD3 levels (Total). **(F)** The association between TG/HDL and DM at different VD3 levels (Separate). TG/HDL, triglycerides/high-density lipoprotein; IR, insulin resistance; VD3, vitamin D3; IGT, impaired glucose tolerance; DM, diabetes mellitus.

Here, we analyzed the non-linear relationships of TG/HDL ratio with IR, IGT, and DM at different VD3 levels. Using the fully adjusted Model 4, we discovered different relationships among VD3 groups. The correlation was fitted by logistic regression (**Figure 4**), and the non-linear relationship was approved by double segmentation (**Table 4**). In addition, *p* < 0.05 was obtained upon the log-likelihood ratio test. Thus, two-stage logistic regression was conducted to accurately describe the relationships of TG/HDL ratio with IR, IGT, and DM at different VD3 levels (**Figure 4** and **Table 4**).

**Table 4:** Threshold analysis of TG/HDL on the incident of IR, IGT, and DM in the NHANES study, 2009–2018.

Outcomes	N1 (VD3 < 30)		N2 (30 ≤ VD3 < 50)		N3 (50 ≤ VD3 < 80)		N4 (80 ≤ VD3)	
	OR (95% CI)	p-Value	OR (95% CI)	p-Value	OR (95% CI)	p-Value	OR (95% CI)	p-Value
**IR group**								
Cutoff value	1.16 (1.14, 1.18)		1.10 (1.08, 1.12)		1.34 (1.31, 1.37)		0.91 (0.88, 0.93)	
TG/HDL < 1.094	5.68 (2.64, 12.21)	<0.001	5.55 (3.27, 9.42)	<0.001	3.50 (2.53, 4.84)	<0.001	9.55 (3.79, 24.06)	<0.001
TG/HDL ≥ 1.094	1.49 (0.89, 2.48)	0.123	1.41 (1.03~1.93)	0.033	1.54 (1.12, 2.11)	0.007	1.84 (1.33, 2.54)	<0.001
Likelihood ratio test	–	<0.001	–	<0.001	–	<0.001	–	<0.001
Non-linear test	–	<0.001	–	<0.001	–	<0.001	–	<0.001
**Prediabetes group**								
Cutoff value	1.46 (1.43, 1.49)		1.22 (1.19, 1.24)		1.11 (1.09, 1.13)		1.97 (1.93, 2.01)	
TG/HDL < 1.51	5.29 (2.94, 9.49)	<0.001	2.68 (1.61, 4.45)	<0.001	2.14 (1.32, 3.48)	<0.001	1.91 (1.40, 2.61)	<0.001
TG/HDL ≥ 1.51	1.13 (0.63, 2.01)	0.685	1.20 (0.85, 1.68)	0.296	1.40 (1.10, 1.78)	0.007	0.06 (0.01, 0.46)	<0.001
Likelihood Ratio test	–	<0.001	–	<0.001	–	<0.001	–	<0.001
Non-linear test	–	<0.001	–	<0.001	–	<0.001	–	<0.001
**Diabetes group**								
Cutoff value	1.18 (1.10, 1.14)		0.82 (0.75, 0.89)		0.82 (0.75, 0.89)		1.94 (1.91, 1.97)	
TG/HDL < 1.11	7.33 (2.43, 22.05)	<0.001	3.83 (1.04, 14.18)	<0.001	7.13 (2.30, 22.08)	<0.001	1.33 (0.99, 1.77)	0.057
TG/HDL ≥ 1.11	1.20 (0.77, 1.85)	0.421	1.53 (1.19, 1.97)	<0.001	1.57 (1.29, 1.94)	<0.001	0.54 (0.11, 2.65)	0.444
Likelihood ratio test	–	<0.001	–	<0.001	–	<0.001	–	<0.001
Non-linear test	–	<0.001	–	<0.001	–	<0.001	–	<0.001

Adjusted for age, gender, race, education, BMI, WC (waist circumference), income, smoking, alcohol use, hypertension, UA, ALT, AST, GGT, LDH, and BUN.

TG/HDL, triglycerides/high-density lipoprotein; IR, insulin resistance; IGT, impaired glucose tolerance; DM, diabetes mellitus; VD3, vitamin D3; BMI, body mass index; UA, uric acid; ALT, alanine aminotransferase; AST, aspartate aminotransferase; GGT, γ-glutamyl transpeptidase; LDH, low-density lipoprotein; BUN, blood urea nitrogen.

#### 4.4.1 Insulin Resistance Group

Based on the two-stage logistic regression models and recursive algorithm, the cutoff value was determined to be 1.094. On the left side of the cutoff value, the ORs and 95% CIs were 5.68 (2.64–12.21), 5.55 (3.27–9.42), 3.50 (2.53–4.84), and 9.55 (3.79–24.06) in the N1–N4 groups, respectively. On the right side of the cutoff value, the ORs and 95% CIs were 1.49 (0.89, 2.48), 1.41 (1.03, 1.93), 1.54 (1.12, 2.11), and 1.84 (1.33, 2.54) in the N1–N4 groups, separately.

#### 4.4.2 Impaired Glucose Tolerance Group

According to the two-stage logistic regression models and recursive algorithm, the cutoff value was set to 1.54. On the left side of the cutoff value, the ORs and 95% CIs were 5.29 (2.94–9.49), 2.68 (1.61–4.45), 2.14 (1.32–3.68), and 1.91 (1.40–2.61) in the N1–N4 groups, respectively. On the right side of the cutoff value, the ORs and 95% CIs were 1.13 (0.63, 2.01), 1.2 (0.85, 1.68), 1.40 (1.1, 1.71), and 0.06 (0.01, 0.46) in the N1–N4 groups, separately.

#### 4.4.3 Diabetes Mellitus Group

In line with the two-stage logistic regression models and recursive algorithm, the cutoff value was 1.11. On the left side of the cutoff value, the ORs and 95% CIs were 7.33 (2.43–22.05), 3.83 (1.04–14.18), 7.13 (2.30–22.08), and 1.33 (0.99–1.77) in the N1–N4 groups, respectively. On the right side of the cutoff value, the ORs and 95% CIs were 1.20 (0.77, 1.85), 1.53 (1.19, 1.97), 1.57 (1.29, 1.94), and 0.54 (0.11, 2.65) in the N1–N4 groups, respectively.

## 5 Discussion

In this study, 5,960 participants were recruited to analyze the independent associations of TG/HDL ratio with IR, IGT, and DM at different VD3 levels. After the influencing factors were adjusted, TG/HDL ratio was related to IR, IGT, and DM at varying degrees at different VD3 levels (*p* for interaction <0.05). Specifically, stronger relationships were observed in the N1 and N4 groups (**Table 3**). It suggests that the too low or too high VD3 levels possibly strengthen the associations of TG/HDL ratio with IR, IGT, and DM.

In a study carried out on Hispanic and African Americans, the associations of TG/HDL ratio with IR, β-cell function, and DM were investigated. After the influencing factors were adjusted, TG/HDL ratio was associated with IR in the non-Caucasian populations, and a higher TG/HDL ratio was related to lower insulin sensitivity in the Hispanic and African American populations (25). Wang et al. also confirmed that TG/HDL ratio was an independent risk factor for DM in the Singapore Chinese (26). In addition, Gong et al. studied more than 100,000 Chinese cohorts and discovered that a higher TG/HDL ratio was positively correlated with the occurrence of IGT and DM after the influencing factors were adjusted (27). These investigations are consistent with our results.

This study further evaluated the effect of VD3 levels on the relationships of TG/HDL ratio with IR, IGT, and DM. As a result, too high or too low VD3 levels promoted the abovementioned relationships. The N3 group (ordinary VD3 levels) showed the weakest correlations of TG/HDL with IR, IGT, and DM. This indicates that, compared with VD3 abundance and deficiency, maintaining the reasonable VD3 levels is more effective in reducing the associations of TG/HDL ratio with IR, IGT, and DM. In addition, we found that such associations were strengthened, not weakened as expected, in the N4 group. This demonstrates that excessive (high level of) VD3 strengthens the associations of TG/HDL with IR, IGT, and DM. The main reason is that excessive VD3 can lead to abnormalities in calcium and phosphorus metabolism as well as VD3 toxicity in the body, thus lowering the protective effect of VD3 on the body. Therefore, this should be further validated by more clinical studies and randomized controlled trials (RCTs).

As reported in previous studies, high TG/HDL ratios lead to reduced retention of fatty acids; therefore, more fatty acids are transported to the liver for TG synthesis, and more free fatty acids will be formed accordingly (28), while higher free fatty acid levels have been identified as a risk factor for T2DM. By combining with insulin, the excess free fatty acids prevent the secretion of a normal amount of insulin from achieving the desired glucose-lowering effect. As a result, pancreatic β-cells are stimulated to secrete more insulin, which thus leads to IR and ultimately the development of DM (29). In addition, TG-rich lipoproteins accelerate the production of leptin, angiotensinogen, tumor necrosis factor-alpha (TNF-a), interleukin 6 (IL-6), fibrinogen activator inhibitor 1, transforming growth factor B (TGF-b), lipocalin, and resistin (29). These factors are the risk factors for the development of IR or DM, at least at the experimental level. VD3 can bind to some free fatty acids (30) to alleviate certain unfavorable effects of fatty acids. This was also represented in results from the N1–N3 groups, where the associations of TG/HDL ratio with IR, IGT, and DM were weakened as VD3 levels increased (**Tables 3** and **4**). As a kind of fat-soluble vitamin, VD3 is mainly obtained by irradiating the skin with sunlight (31). Studies have reported that phototherapy and VD3 supplementation can ameliorate IR and inflammation in a rat model of non-alcoholic fatty liver disease (NAFLD) induced by a special diet (32). Based on these results and our observation, we suppose that VD3 is beneficial to normal metabolism, and safe sunlight exposure can be an appropriate way to increase VD3 levels for human health (33).

Certainly, some of the results in this study were different from those of previous studies. Such discrepancies may be explained from the following aspects. 1) The study populations were different. 2) The adjusted variables were different, and a more adequate adjustment strategy was adopted in this study. 3) The associations of TG/HDL ratio with IR, IGT, and DM were analyzed at different VD3 levels in this study. Notably, the clinical strength of this study is as follows. We found that within a reasonable range of VD3 (0–80 ng/ml), increasing VD3 levels attenuated the associations of TG/HDL ratio with IR, IGT, and DM. This provides new evidence for clinical guidance on the appropriate range of VD3 levels in patients. Meanwhile, we discovered that when VD3 levels were too high (≥80 ng/ml) in DM patients, it might strengthen the relations of TG/HDL ratio with IR, IGT, and DM. This indicates that the incidence of abnormal glucose metabolism may increase in people with high TG/HDL ratios when VD3 levels are too high.

Nevertheless, certain limitations should also be noted in this study. 1) This was a cross-sectional study, and the causal relationships of TG/HDL ratio with IR, IGT, and DM were not determined. Therefore, a cohort study is warranted to analyze the accurate relationship. 2) Special populations (like pregnant women and children) were excluded from this study, and whether the results were applicable to these populations remains unknown. Also, there are some noteworthy highlights of this study. First, this study was conducted using the official NHANES database, which is more representative of the entire US population after a complex weighting design. Second, in the design of this study, smoothed fitting curve and two-stage logistic regression were used to accurately analyze the relationships.

## 6 Conclusion

Collectively, our results suggest that in the American population, maintaining too high or too low levels of VD3 can promote the associations of TG/HDL with IR, IGT, and DM, which shed new light on DM research. However, other conditions such as age and sunlight exposure level should be taken into consideration when formulating the appropriate VD3 levels.

## Data Availability Statement

Publicly available datasets were analyzed in this study. Data can be obtained from the NHANES database (https://www.cdc.gov/nchs/nhanes/).

## Author Contributions

YL and RG: conceived the idea; YL, RG and GL wrote the manuscript; GL and RG collected and read the literature and revised the article; XW and LY read through and corrected the manuscript. All authors contributed to the article and approved the submitted version. YL is the first author. RG and GL are the co-first author. XW and LY are the corresponding author of this paper.

## Funding

This work was supported by Grants from the National Key R&D Program of China (2018YFC1311500), the National Natural Science Foundation of China (81860370), the CAS “Light of West China” Program (2019), the General Project of Natural Science Foundation of Qinghai Province (2020-ZJ-930) Effects of high altitude environment and season on vitamin D, bone metabolism factors and muscle volume in adults.

## Conflict of Interest

The authors declare that the research was conducted in the absence of any commercial or financial relationships that could be construed as a potential conflict of interest.

## Publisher’s Note

All claims expressed in this article are solely those of the authors and do not necessarily represent those of their affiliated organizations, or those of the publisher, the editors and the reviewers. Any product that may be evaluated in this article, or claim that may be made by its manufacturer, is not guaranteed or endorsed by the publisher.

## Abbreviations

IR, insulin resistance; IGT, impaired glucose tolerance; DM, diabetes mellitus; EDU, education; SMOK, smoking; BMI, body mass index; ALT, alanine aminotransferase; ALB, albumin; AST, aspartate aminotransferase; ALP, alkaline phosphatase; BUN, blood urea nitrogen; GGT, γ-glutamyl transpeptidase; UA, uric acid; FPG, fasting plasma glucose; TC, total cholesterol.
